# Exploring the Performance of ChatGPT Versions 3.5, 4, and 4 With Vision in the Chilean Medical Licensing Examination: Observational Study

**DOI:** 10.2196/55048

**Published:** 2024-04-29

**Authors:** Marcos Rojas, Marcelo Rojas, Valentina Burgess, Javier Toro-Pérez, Shima Salehi

**Affiliations:** 1 Graduate School of Education, Stanford University, Stanford, CA, United States; 2 School of Medicine, Universidad de Chile, Santiago, Chile

**Keywords:** artificial intelligence, AI, generative artificial intelligence, medical education, ChatGPT, EUNACOM, medical licensure, medical license, medical licensing exam

## Abstract

**Background:**

The deployment of OpenAI’s ChatGPT-3.5 and its subsequent versions, ChatGPT-4 and ChatGPT-4 With Vision (4V; also known as “GPT-4 Turbo With Vision”), has notably influenced the medical field. Having demonstrated remarkable performance in medical examinations globally, these models show potential for educational applications. However, their effectiveness in non-English contexts, particularly in Chile’s medical licensing examinations—a critical step for medical practitioners in Chile—is less explored. This gap highlights the need to evaluate ChatGPT’s adaptability to diverse linguistic and cultural contexts.

**Objective:**

This study aims to evaluate the performance of ChatGPT versions 3.5, 4, and 4V in the EUNACOM (Examen Único Nacional de Conocimientos de Medicina), a major medical examination in Chile.

**Methods:**

Three official practice drills (540 questions) from the University of Chile, mirroring the EUNACOM’s structure and difficulty, were used to test ChatGPT versions 3.5, 4, and 4V. The 3 ChatGPT versions were provided 3 attempts for each drill. Responses to questions during each attempt were systematically categorized and analyzed to assess their accuracy rate.

**Results:**

All versions of ChatGPT passed the EUNACOM drills. Specifically, versions 4 and 4V outperformed version 3.5, achieving average accuracy rates of 79.32% and 78.83%, respectively, compared to 57.53% for version 3.5 (*P*<.001). Version 4V, however, did not outperform version 4 (*P*=.73), despite the additional visual capabilities. We also evaluated ChatGPT’s performance in different medical areas of the EUNACOM and found that versions 4 and 4V consistently outperformed version 3.5. Across the different medical areas, version 3.5 displayed the highest accuracy in psychiatry (69.84%), while versions 4 and 4V achieved the highest accuracy in surgery (90.00% and 86.11%, respectively). Versions 3.5 and 4 had the lowest performance in internal medicine (52.74% and 75.62%, respectively), while version 4V had the lowest performance in public health (74.07%).

**Conclusions:**

This study reveals ChatGPT’s ability to pass the EUNACOM, with distinct proficiencies across versions 3.5, 4, and 4V. Notably, advancements in artificial intelligence (AI) have not significantly led to enhancements in performance on image-based questions. The variations in proficiency across medical fields suggest the need for more nuanced AI training. Additionally, the study underscores the importance of exploring innovative approaches to using AI to augment human cognition and enhance the learning process. Such advancements have the potential to significantly influence medical education, fostering not only knowledge acquisition but also the development of critical thinking and problem-solving skills among health care professionals.

## Introduction

The launch of OpenAI’s ChatGPT-3.5 in November 2022 has impacted various fields, including medical education [1]. On September 25, 2023, OpenAI announced the release of a highly anticipated new functionality, ChatGPT-4 With Vision (4V; also known as “GPT-4 Turbo With Vision”), to support multimodal interaction and further exploration [2].

ChatGPT has shown promise, or some would argue that it is a threat, for medical education with its outstanding performance in several medical examinations. For example, in the Médicos Internos Residentes examination in Spain [3], ChatGPT answered 51.4% of the questions correctly [3]. In the United States, different studies have reported an accuracy of 80%-90% on the United States Medical Licensing Examination [4]. These results highlight ChatGPT’s potential to impact the future of medical education. However, there is a limited understanding of ChatGPT’s performance in non-English examinations in Latin America, such as Chile’s EUNACOM (Examen Único Nacional de Conocimientos de Medicina).

The EUNACOM comprises 180 multiple-choice questions from various medical areas such as internal medicine, pediatrics, obstetrics and gynecology, surgery (general surgery and anesthesia, traumatology, and urology), psychiatry, specialties (including dermatology, ophthalmology, and otorhinolaryngology), and public health. The examination assesses topics such as diagnosis, treatment, and follow-up care. Passing the EUNACOM is vital for foreign-trained doctors to practice in Chile and for Chilean medical students to complete their studies and transition to medical practice [5]. This examination, central to Chilean medical education, can potentially pose linguistic, cultural, and contextual challenges to ChatGPT. This study aimed to evaluate the performance of ChatGPT versions 3.5, 4, and the recently released 4V on EUNACOM practice drills, with the intent to guide future improvements—specifically, the integration and use of artificial intelligence (AI) in medical education—across various cultural and linguistic contexts, thereby contributing to the ongoing debate on the role and efficacy of AI as an educational tool in the global medical community.

## Methods

### Study Design

We adopted a quantitative, descriptive, cross-sectional approach to evaluate ChatGPT’s performance in the EUNACOM practice drills. We gathered a data set of EUNACOM practice questions, categorized them, and analyzed the responses of ChatGPT versions 3.5, 4, and 4V.

### EUNACOM Data Set

It is challenging to obtain an authentic and representative set of questions from the EUNACOM, as the examination is not publicly accessible for integrity and security reasons. Therefore, we used 3 official practice drills designed by the University of Chile as preparatory material for its students. These drills are not included in the data used to train ChatGPT due to their limited public availability. Each drill consists of 180 multiple-choice questions with 5 options, where only 1 is correct. The number of questions across medical areas in each drill reflects the specifications of the EUNACOM’s administrative office (ie, internal medicine, n=67; pediatrics, n=29; obstetrics and gynecology, n=29; surgery, n=20; psychiatry, n=14; specialties, n=12; and public health, n=9).

### Classification of Questions

The categorization of EUNACOM’s questions in this study is in line with that of Carrasco et al [3] in 2023 on the Médicos Internos Residentes examination in Spain. Two of our research team members classified the questions as follows:

1. Medical area according to the EUNACOM: internal medicine, pediatrics, obstetrics and gynecology, surgery, psychiatry, specialties, and public health.
2. Category of questions: “clinical case” if they presented a clinical case in the stem of the question, or “medical knowledge” if the question asked for the retrieval of knowledge of medical content.
3. Type of question in clinical case questions: diagnosis, treatment, or follow-up.

### Prompting and Application of ChatGPT

We used ChatGPT versions 3.5, 4, and 4V, trained up to January 2022, to respond to the 3 EUNACOM drills in October 2023. Each drill was conducted 3 times with each version of ChatGPT using the prompt, “Which is the correct answer to the following questions?” We excluded “EUNACOM” from the prompt to guarantee ChatGPT’s responsiveness to the questions, since, according to OpenAI’s policies, the model abstains from taking official assessments. When using version 4V, we prompted questions with images (eg, x-ray) individually, attaching the image to its corresponding question.

The 3 attempts at providing responses in each drill allowed us to address the variability in ChatGPT’s answers, attributable to its probabilistic nature, by estimating an average performance.

### Data Analysis

Data analysis was conducted using Stata (version 16.0; StataCorp). We computed the percentage of correct responses for each drill and set the passing score at >51% in accordance with the EUNACOM standard [6]. We used a 2-sample test of proportions to test for differences in performance among different versions of ChatGPT [7].

### Ethical Considerations

The Human Research Ethics Committee of the Faculty of Medicine at the University of Chile determined that this study presented no ethical concerns that warranted institutional review board oversight. We used EUNACOM drills authorized by the University of Chile’s School of Medicine because access to the actual examination is restricted.

## Results

The three versions of ChatGPT successfully passed EUNACOM drills on average. Notably, version 4 exhibited superior performance to that of version 3.5 across all drills and attempts, while version 4V did not show a statistically significant advantage over version 4. The only instance of not passing the EUNACOM was observed with version 3.5, specifically during its third attempt at drill 2. Detailed performance metrics for each drill and attempt are provided in Table 1. To assess the robustness of our results, we also compared the performance of ChatGPT by each attempt and by each drill. The results are qualitatively similar.

**Table 1.** Correct answers of ChatGPT versions 3.5, 4, and 4 With Vision on each of the EUNACOM^a^ drills (each with 180 multiple-choice questions) per attempt.

EUNACOM drill and attempt		Correct answers provided by each version of ChatGPT, n (%)		
		3.5^b^	4^c^	4 With Vision^d^
**Drill 1**				
	1	105 (58.33)	143 (79.44)	147 (81.67)
	2	109 (60.56)	148 (82.22)	149 (82.78)
	3	103 (57.22)	146 (81.11)	145 (80.56)
**Drill 2**				
	1	93 (51.67)	138 (76.67)	133 (73.89)
	2	94 (52.22)	134 (74.44)	139 (77.22)
	3	86 (47.78)^e^	132 (73.33)	137 (76.11)
**Drill 3**				
	1	112 (62.22)	143 (79.44)	142 (78.89)
	2	114 (63.33)	150 (83.33)	139 (77.22)
	3	116 (64.44)	151 (83.89)	146 (81.11)

a EUNACOM: Examen Único Nacional de Conocimientos de Medicina.

b Mean accuracy rate 57.53% (95% CI 55.12%-59.94%).

c Mean accuracy rate 79.32% (95% CI 77.35%-81.29%); *z*_3.5 vs 4_=–13.34, *P*<.001 (2-sample test of proportions).

d Mean accuracy rate 78.83% (95% CI 76.84%-80.82%); *z*_4 vs 4V_=0.35, *P*=.73 (2-sample test of proportions).

e This is the only instance of not passing the EUNACOM practice drill.

Across all attempts and the 3 practice drills, we observed a variation in average accuracy rates by both medical area and clinical case question type. In an evaluation across various medical areas, all 3 ChatGPT versions demonstrated distinct high and low performances. For version 3.5, the highest accuracy was observed in psychiatry (average 69.84%), while the lowest accuracy rate was observed in internal medicine (average 52.74%). Version 4 excelled in surgery with a 90.00% average accuracy rate, whereas its weakest performance was observed in internal medicine (average 75.62%). Similarly, version 4V’s performance was strongest in surgery (average 86.11%) and weakest in public health (average 74.07%). When analyzing performance across different medical areas, ChatGPT-4 consistently outperformed ChatGPT-3.5. However, ChatGPT-4V did not significantly outperform ChatGPT-4.

The 3 drills included a total of 501 clinical case questions and 39 medical knowledge questions. In answering clinical case questions, the average accuracy rate of ChatGPT across the 3 attempts was as follows: 57.22% for version 3.5, 80.11% for version 4, and 79.71% for version 4V. In answering medical knowledge questions, the average accuracy rate of ChatGPT was as follows: 61.54% for version 3.5, 74.36% for version 4, and 67.52% for version 4V.

Among the clinical case questions, ChatGPT performed best in follow-up questions, with version 4 scoring 88.89%, while the lowest performance was observed in treatment questions, with version 3.5 scoring 48.50%. On analyzing performance over different types of clinical case questions, ChatGPT-4 regularly outperformed ChatGPT-3.5. Nonetheless, ChatGPT-4V showed no significant difference in performance compared to ChatGPT-4. Comprehensive data on average performances across all medical areas and types of clinical case questions are included in Multimedia Appendix 1.

The 3 drills had a total of 50 questions with images; therein, ChatGPT-4 had an average accuracy rate of 70.67% and version 4V had an average accuracy rate of 70.00% across the 3 attempts.

## Discussion

### Principal Findings

This study shows that ChatGPT successfully passed the EUNACOM, with version 4 showing a superior performance to that of version to 3.5. However, interestingly, version 4V did not significantly outperform version 4 in this examination. All versions demonstrated proficiency in various medical specialties, with version 3.5 excelling in psychiatry and versions 4 and 4V in surgery. However, unexpectedly, version 4V did not outperform the other 2 versions in questions including images. The differences in performance among versions are likely due to continuous enhancements in training and knowledge with each update, which improve the models’ grasp of complex medical subjects. Nevertheless, varying success rates in specific medical fields could stem from the complexities of those specialties, unique terminologies, or the specific structure of the questions in those areas, which may align differently with the data the models were trained on.

In particular, when analyzing the question categories, all versions presented a lower accuracy rate in medical knowledge questions than in clinical case questions, indicating a possible gap in the models’ data regarding specific content knowledge. In clinical case questions, versions 4 and 4V consistently outperformed version 3.5, possibly due to the AI’s advancement in pattern recognition. Interestingly, each version performed differently across various types of questions in the clinical case category: version 3.5 showed a lower performance on treatment and follow-up questions, whereas versions 4 and 4V performed better on follow-up questions, suggesting an enhanced ability to handle dynamic, evolving clinical scenarios in later versions.

The modest enhancements in visual data interpretation from ChatGPT-4 to ChatGPT-4V indicate that improvements in later versions focused more on specific refinements rather than on broad upgrades to support image processing. This trend is evident in image-based questions, where version 4V did not outperform version 4 in questions including images. For example, while ChatGPT showed improved accuracy in interpreting electrocardiograms, its performance was less consistent with dermatological images. A striking instance was its misdiagnosis of a *Staphylococcus aureus* skin infection in a toddler, where ChatGPT incorrectly identified the condition as Molluscum contagiosum, erroneously attributing significance to an area of the image that was, in fact, the patient’s belly button. These variations underscore the intricate challenges AI faces in processing multimodal medical information and suggest that while ChatGPT’s textual understanding has advanced, its image processing requires further contextual depth and fine-tuning.

ChatGPT’s strong performance on medical licensing examinations from different parts of the world and in different languages demonstrates its adaptability and potential in medical education despite not being specifically designed for such specialized content [3,4,8-10]. However, its varying responses highlight the model’s limitations in handling the depth and variability of real-life medical expertise.

This study is one of the first to evaluate ChatGPT-4, including its vision-enhanced iteration, in medical licensing examinations, notably being the first to evaluate its performance in Chile’s EUNACOM. The multiple attempts per practice drill approach in our methodology is a significant strength of our study, facilitating a thorough examination of ChatGPT’s response consistency. Despite these strengths, the study has some limitations. The reliance on practice drills from the University of Chile may not encompass the full breadth of the EUNACOM’s questions, potentially narrowing the scope of our findings. The focus on specific versions of ChatGPT could also limit the generalizability of our results to other iterations of the model. Inherent biases in the AI’s training data pose another challenge, potentially affecting the accuracy of responses.

Future studies should expand AI evaluations in medical training by including diverse medical examinations and question types, assessing adaptability to various contexts. Exploring newer AI models and their performance in practical medical scenarios will also be crucial. This research will enhance the understanding of AI’s role in medicine, guiding its effective integration into health care education and practice.

The rise of generative AI in medicine, highlighted by tools such as ChatGPT and upcoming models such as Med-PaLM [11], signals a need to evolve medical education. While these tools provide extensive resources, the essence of medical practice extends beyond simple access to data, necessitating reflective and critical application of this knowledge. Therefore, medical curricula must prioritize critical thinking, enabling future practitioners to discern the quality and relevance of AI-generated information. Similarly, reflective practices are crucial, promoting continuous self-assessment and adaptation in a rapidly advancing technological landscape. As AI becomes integral, especially in diagnostics, professionals must merge AI insights with human-centric care, underscoring that medical expertise is not only about accessing information but also involves deep understanding and evaluation of that information, empathy, and ethical judgment.

### Conclusions

In conclusion, this study shows the performance of ChatGPT versions 3.5, 4, and 4V in successfully passing the EUNACOM, underscoring the evolving role of AI in the field of medicine and its potential in medical education. Future studies should encompass a wider array of AI models and diverse question types, contributing to a deeper understanding of how AI can enhance medical education. Moreover, it is imperative to explore innovative directions in the application of AI, such as leveraging AI to augment human cognition and optimize the learning process. Embracing these possibilities can lead to a more profound impact on medical education, fostering not only knowledge acquisition but also critical thinking and problem-solving skills among future health care practitioners.

## Supplementary material

10.2196/55048 Multimedia Appendix 1 Average accuracy rate per medical area and clinical case question type.

## Abbreviations

4V: ChatGPT-4 With Vision
AI: artificial intelligence
EUNACOM: Examen Único Nacional de Conocimientos de Medicina
