# Supplementary material for: Exploring the Performance of ChatGPT Versions 3.5, 4, and 4 With Vision in the Chilean Medical Licensing Examination: Observational Study
Source: JMIR Med Educ. 2024 Apr 29;10:e55048. doi: 10.2196/55048 (PMC11082432; doi:10.2196/55048)
Supplement: Multimedia Appendix 1 [file mededu-v10-e55048-s001.docx]

## Appendix 1 Average accuracy rate per medical area and types of clinical case questions

Table 1. Average accuracy rate per medical area for ChatGPT versions 3.5, 4, and 4V, based on 540 unique questions from the three EUNACOM drills.

| **Medical area** | **Number of unique questions** | **Average accuracy rate by version of ChatGPT across three attempts** | | |
| --- | --- | --- | --- | --- |
|  |  | **ChatGPT 3.5, %** | **ChatGPT 4, % (*P* value)^a^** | **ChatGPT 4V, % (*P* value)^b^** |
| Internal medicine | 201 | 52.74 | 75.62 (*P*<.001) | 78.11 (*P*=.221) |
| Pediatrics | 87 | 57.85 | 81.61 (*P*<.001) | 78.91 (*P*=.663) |
| Gynecology-Obstetrics | 87 | 56.70 | 77.78 (*P*<.001) | 74.33 (*P*=.356) |
| Surgery | 60 | 62.22 | 90.00 (*P*<.001) | 86.11 (*P*=.255) |
| Psychiatry | 42 | 69.84 | 82.54 (*P*=.018) | 82.54 (*P*=1.000) |
| Specialties | 36 | 58.33 | 79.63 (*P*=.001) | 80.56 (*P*=.865) |
| Public Health | 27 | 64.20 | 82.72 (*P*=.008) | 74.07 (*P*=.181) |

^a^This *P* value is calculated from a significance test comparing the performance of ChatGPT 3.5 and ChatGPT 4.

^b^This *P* value is calculated from a significance test comparing the performance of ChatGPT 4 and ChatGPT 4V.

Table 2. Average accuracy rate per types of clinical case questions for ChatGPT versions 3.5, 4, and 4V, based on 501 unique Clinical case questions from the three EUNACOM drills.

| **Type of question** | **Number of unique questions** | **Average accuracy rate by version of ChatGPT across three attempts** | | |
| --- | --- | --- | --- | --- |
|  |  | **ChatGPT 3.5, %** | **ChatGPT 4, % (*P* value)^a^** | **ChatGPT 4V, % (*P* value)^b^** |
| Diagnosis | 308 | 62.55 | 81.60 (*P*<.001) | 83.12 (*P*=.274) |
| Treatment | 178 | 48.50 | 76.78 (*P*<.001) | 73.41 (*P*=.259) |
| Follow-up | 15 | 51.11 | 88.89 (*P*<.001) | 84.44 (*P*=.535) |

^a^This *P* value is calculated from a significance test comparing the performance of ChatGPT 3.5 and ChatGPT 4.

^b^This *P* value is calculated from a significance test comparing the performance of ChatGPT 4 and ChatGPT 4V.
